# Supplementary material for: Density of Aedes aegypti and dengue virus transmission risk in two municipalities of Northwestern Antioquia, Colombia
Source: PLoS One. 2024 Jan 25;19(1):e0295317. doi: 10.1371/journal.pone.0295317 (PMC10810462; doi:10.1371/journal.pone.0295317)
Supplement: Attachment — Submitted filename: PONE-D-23-22160_reviewer_Reviewed.pdf [file pone.0295317.s002.pdf]

PLOS ONE

Density of Aedes aegypti and Dengue Virus Transmission Risk in Two Municipalities of Northwestern Antioquia, Colombia

--Manuscript Draft--

Manuscript Number:	PONE-D-23-22160
Article Type:	Research Article
Full Title:	Density of Aedes aegypti and Dengue Virus Transmission Risk in Two Municipalities of Northwestern Antioquia, Colombia
Short Title:	Aedes aegypti density and dengue risk in Northwestern Antioquia, Colombia
Corresponding Author:	Wilber Gómez-Vargas, M.D<br>CES University: Universidad CES<br>Medelllín, Antioquia COLOMBIA
Keywords:	Aedes aegypti; vector control; Dengue; Aedic indices; Transmission risk; RT-PCR.
Abstract:	The high infestation of Aedes aegypti populations in Urabá, Antioquia, Colombia represents a risk factor for increased dengue morbidity and mortality. This study aimed to determine the risk of dengue transmission by estimating the population of Ae. aegypti using entomological indices, density of females per dwelling and inhabitant, and virological surveillance in two municipalities in Colombia. A cross-sectional study was conducted with quarterly entomological surveys in three neighborhoods of Apartadó and Turbo between 2021 and 2022. Aedic indices and vector density per dwelling and per inhabitant were calculated. The Kernel method was used for spatial analysis, and correlations between climatic variables and mosquito density were examined. Virus detection and serotyping in mosquitoes was performed using single-step reverse transcription polymerase chain reaction. The housing, reservoir, and Breteau indices were 48.9 %, 29.5 %, and 70.2 %, respectively. The mean density of Ae. aegypti was 1.47 females / dwelling and 0.51 females / inhabitant. The overall visual analysis showed several critical points in the neighborhoods studied. There was significant correlation of vector density and relative humidity and precipitation in the neighborhoods 29 de noviembre and 24 de diciembre. Additionally, serotypes DENV-1 and DENV-2 were found. The overall indices for dwellings, reservoirs, and Breteau were lower than those recorded in 2014 in Urabá. The vector density results in this study were similar to those reported in other studies conducted in Latin America, and vector infection was detected. The aedic and density indices are complementary, emphasizing the importance of continuous surveillance of Ae. aegypti to inform appropriate control strategies and prevent future dengue outbreaks in these municipalities.
Order of Authors:	Wilber Gómez-Vargas, PhD student
	Paola Astrid Ríos Tapias, Master in epidemiology
	Katerine Marín-Velásquez, Master in epidemiology
	Erika Giraldo-Gallo, PhD
	Angela Segura-Cardona, PhD
	Margarita Arboleda, PhD
Additional Information:	
Question	Response
**Financial Disclosure**<br><br>Enter a financial disclosure statement that describes the sources of funding for the work included in this submission. Review the submission guidelines for detailed requirements. View published research	This work was financially supported by the Ministry of Science, Technology, and Innovation of Colombia under project code 67882. The funder had no role in study design, data collection<br>and analysis, decision to publish, or preparation of the manuscript.

articles from *PLOS ONE* for specific examples.<br><br>This statement is required for submission and **will appear in the published article** if the submission is accepted. Please make sure it is accurate.<br><br>**Unfunded studies**<br>Enter: *The author(s) received no specific funding for this work.*<br><br>**Funded studies**<br>Enter a statement with the following details:<br>• Initials of the authors who received each award<br>• Grant numbers awarded to each author<br>• The full name of each funder<br>• URL of each funder website<br>• Did the sponsors or funders play any role in the study design, data collection and analysis, decision to publish, or preparation of the manuscript?<br>• **NO** - Include this sentence at the end of your statement: *The funders had no role in study design, data collection and analysis, decision to publish, or preparation of the manuscript.*<br>• **YES** - Specify the role(s) played.<br><br>* typeset	
**Competing Interests**<br><br>Use the instructions below to enter a competing interest statement for this submission. On behalf of all authors, disclose any competing interests that could be perceived to bias this work—acknowledging all financial support and any other relevant financial or non-financial competing interests.<br><br>This statement is required for submission and **will appear in the published article** if the submission is accepted. Please make sure it is accurate and that any funding sources listed in your Funding Information later in the submission form are also declared in your Financial Disclosure statement.	The authors have declared that no competing interests exist.

View published research articles from *PLOS ONE* for specific examples.<br><br>**NO authors have competing interests**<br><br>Enter: *The authors have declared that no competing interests exist.*<br><br>**Authors with competing interests**<br><br>Enter competing interest details beginning with this statement:<br><br>*I have read the journal's policy and the authors of this manuscript have the following competing interests: [insert competing interests here]*<br><br>* typeset	
**Ethics Statement**<br><br>Enter an ethics statement for this submission. This statement is required if the study involved:<br><br>• Human participants<br>• Human specimens or tissue<br>• Vertebrate animals or cephalopods<br>• Vertebrate embryos or tissues<br>• Field research<br><br>Write "N/A" if the submission does not require an ethics statement.<br><br>General guidance is provided below. Consult the submission guidelines for detailed instructions. **Make sure that all information entered here is included in the Methods section of the manuscript.**	The research protocol was submitted to the Institutional Bioethics Committee of the Instituto Colombiano de Medicina Tropical-Universidad CES in Medellín, and it received approval under Act 66 on June 14, 2019.

**Format for specific study types**<br><br>**Human Subject Research (involving human participants and/or tissue)**<br>• Give the name of the institutional review board or ethics committee that approved the study<br>• Include the approval number and/or a statement indicating approval of this research<br>• Indicate the form of consent obtained (written/oral) or the reason that consent was not obtained (e.g. the data were analyzed anonymously)<br><br>**Animal Research (involving vertebrate animals, embryos or tissues)**<br>• Provide the name of the Institutional Animal Care and Use Committee (IACUC) or other relevant ethics board that reviewed the study protocol, and indicate whether they approved this research or granted a formal waiver of ethical approval<br>• Include an approval number if one was obtained<br>• If the study involved *non-human primates*, add *additional details* about animal welfare and steps taken to ameliorate suffering<br>• If anesthesia, euthanasia, or any kind of animal sacrifice is part of the study, include briefly which substances and/or methods were applied<br><br>**Field Research**<br><br>Include the following details if this study involves the collection of plant, animal, or other materials from a natural setting:<br>• Field permit number<br>• Name of the institution or relevant body that granted permission	
**Data Availability**<br><br>Authors are required to make all data underlying the findings described fully available, without restriction, and from the time of publication. PLOS allows rare exceptions to address legal and ethical concerns. See the PLOS Data Policy and FAQ for detailed information.	Yes - all data are fully available without restriction

A Data Availability Statement describing where the data can be found is required at submission. Your answers to this question constitute the Data Availability Statement and **will be published in the article**, if accepted.<br><br>**Important:** Stating 'data available on request from the author' is not sufficient. If your data are only available upon request, select 'No' for the first question and explain your exceptional situation in the text box.<br><br>Do the authors confirm that all data underlying the findings described in their manuscript are fully available without restriction?	
**Describe where the data may be found in full sentences. If you are copying our sample text, replace any instances of XXX with the appropriate details.**<br><br>• If the data are **held or will be held in a public repository**, include URLs, accession numbers or DOIs. If this information will only be available after acceptance, indicate this by ticking the box below. For example: *All XXX files are available from the XXX database (accession number(s) XXX, XXX.*).<br>• If the data are all contained **within the manuscript and/or Supporting Information files**, enter the following: *All relevant data are within the manuscript and its Supporting Information files.*<br>• If neither of these applies but you are able to provide **details of access elsewhere**, with or without limitations, please do so. For example:<br><br>*Data cannot be shared publicly because of [XXX]. Data are available from the XXX Institutional Data Access / Ethics Committee (contact via XXX) for researchers who meet the criteria for access to confidential data.*<br><br>*The data underlying the results presented in the study are available from (include the name of the third party*	All relevant data are within the manuscript and its Supporting Information files.

and contact information or URL).<br>• This text is appropriate if the data are owned by a third party and authors do not have permission to share the data.<br><br>* typeset	
Additional data availability information:	Tick here if the URLs/accession numbers/DOIs will be available only after acceptance of the manuscript for publication so that we can ensure their inclusion before publication.

Powered by Editorial Manager® and ProduXion Manager® from Aries Systems Corporation

Manuscript

Click here to access/download;Manuscript;Density of Aedes aegypti and Dengue Virus Transmission.docx

Density of *Aedes aegypti* and Dengue Virus Transmission Risk in Two Municipalities of Northwestern Antioquia, Colombia

Aedes aegypti density and dengue risk in Northwestern Antioquia, Colombia

Wilber Gómez-Vargas[1*], Paola Astrid Ríos-Tapias[2], Katerine Marin-Velásquez[3], Erika Giraldo-Gallo[1], Angela Segura-Cardona[1], Margarita Arboleda[2]

[1]Epidemiology and Biostatistics Group, Graduate School, Universidad CES, Medellín, Colombia.

[2]Tropical Medicine Group, Colombian Institute of Tropical Medicine - Universidad CES, Apartadó, Colombia

[3]Tropical Medicine Group, Colombian Institute of Tropical Medicine - Universidad CES, Sabaneta, Colombia

[*]gomez.wilber@uces.edu.co (WGV)

Abstract

The high infestation of *Aedes aegypti* populations in Urabá, Antioquia, Colombia represents a risk factor for increased dengue morbidity and mortality. This study aimed to determine the risk of dengue transmission by estimating the population of *Ae. aegypti* using entomological indices, density of females per dwelling and inhabitant, and virological surveillance in two municipalities in Colombia. A cross-sectional study was conducted with quarterly entomological surveys in three neighborhoods of Apartadó and Turbo between 2021 and 2022. Aedic indices and vector density per dwelling and per inhabitant were calculated. The Kernel method was used for spatial analysis, and correlations between climatic variables and mosquito density were examined. Virus detection and serotyping in mosquitoes was performed using single-step reverse transcription polymerase chain reaction. The housing, reservoir, and Breteau indices were 48.9 %, 29.5 %, and 70.2 %, respectively. The mean density of *Ae. aegypti* was 1.47 females / dwelling and 0.51 females / inhabitant. The overall visual analysis showed several critical points in the neighborhoods studied. There was significant correlation of vector density and relative humidity and precipitation in the neighborhoods 29 de noviembre and

24 de diciembre. Additionally, serotypes DENV-1 and DENV-2 were found. The overall indices for dwellings, reservoirs, and Breteau were lower than those recorded in 2014 in Urabá. The vector density results in this study were similar to those reported in other studies conducted in Latin America, and vector infection was detected. The aedic and density indices are complementary, emphasizing the importance of continuous surveillance of *Ae. aegypti* to inform appropriate control strategies and prevent future dengue outbreaks in these municipalities.

Key words: *Aedes aegypti*, Vector control, Dengue, Aedic indices, Transmission risk, RT-PCR.

Introduction

The incidence of dengue and other arboviruses, such as chikungunya and Zika, in Colombia is closely associated with the density of the *Aedes aegypti* vector and its breeding sites, which can be found in urban, peri-urban, and rural areas where stagnant water is present (1,2,3). Consequently, vector control measures remain the primary strategy for preventing dengue outbreaks until specific treatments and effective vaccines become available (4). While *Aedes albopictus*, another

mosquito species, has been found to carry dengue viruses in Colombia, it has not been implicated as a vector thus far (5–7) and there is no record of its presence in the Urabá region.

The Urabá region is particularly vulnerable to *Ae. aegypti* infestation due to its climate and high population density. Unregulated urbanization, coupled with inadequate sanitary conditions and deteriorating infrastructure, creates favorable conditions for the reproduction of *Ae. aegypti*. Although the subregion has achieved approximately 80 % coverage of water and sewerage services, rural areas have significantly lower coverage, with less than 33 % (8). As a result, irregular service interruptions occur, leading to water scarcity during certain times of the year. Inhabitants resort to storing water in various containers, thereby facilitating the breeding of the vector.

Entomological surveillance plays a crucial role in monitoring and controlling *Ae. aegypti* populations. It involves systematic searches for breeding sites containing immature stages (larvae and pupae) as well as adult mosquitoes in urban and peri-urban areas. The objective is to gather information on infestation levels in each locality, serving as a fundamental component of vector control programs (9). By conducting this surveillance, it becomes possible to assess the risk of arbovirus

outbreaks, including dengue, chikungunya, and Zika, in different areas. This information is vital for directing appropriate and timely interventions for vector control (10).

The Colombian Ministry of Health and Social Protection has established the use of entomological or aedic surveys as an official method for assessing the presence and abundance of *Ae. aegypti* (2). These surveys use indices such as the dwelling index, reservoir index, and Breteau index. These indices are based on the number of houses visited and the presence or absence of immature mosquito stages. Despite operational challenges, such as difficulties in accessing residents' houses or locating hidden breeding sites like gutters or sewers, the results obtained through these indices are used to guide vector control efforts (11). However, there are concerns regarding the reliability of these surveys as a method for evaluating *Ae. aegypti* populations. One limitation is that the indices obtained do not take into account the variation in adult mosquito production among different types of containers. Additionally, when vector infestation levels are low, these surveys may not accurately detect the presence of *Ae. aegypti* (12).

Among the various methods used in entomological surveillance, the collection of adult mosquitoes is particularly valuable as it provides

information on the actual number of adult mosquitoes present. This data can be used to calculate the density of *Ae. aegypti* females per unit area or per inhabitant, which is a more accurate predictor of dengue occurrence (13,14). By focusing on adult mosquitoes, this method directly assesses the vector population responsible for disease transmission.

In addition, the use of geographic information systems (GIS) and spatial analysis has become increasingly important in predicting vector dispersion and implementing effective control measures. One useful technique in this regard is kernel density estimation (KDE), which involves smoothing and interpolating the distribution of points across the entire study area. KDE allows for the analysis of focal patterns and provides results that are easily interpretable (15). By incorporating GIS and spatial analysis, researchers and public health authorities can gain valuable insights into the spatial distribution of *Ae. aegypti* populations and make informed decisions regarding vector control strategies.

The Urabá region has been experiencing a predominance of acute febrile syndrome, and dengue infection has been identified as a significant contributor to this syndrome. A previous study conducted in the municipalities of Apartadó, Necoclí, and Turbo revealed a high

frequency of dengue infection, accounting for 37.3 % of non-malarial febrile syndromes among the population (16).

Given the ongoing concerns regarding dengue transmission, the present study aimed to assess several indicators related to dengue risk. This included determining the aedic indices, measuring the density of *Ae. aegypti* mosquitoes, analyzing their spatial distribution, and investigating the correlation between climatic variables (such as temperature, relative humidity, and precipitation) and vector density. Additionally, the study aimed to determine the rate of dengue virus infection. By examining these indicators in three specific neighborhoods of the Apartadó and Turbo municipalities in Antioquia, Colombia, the study aimed to establish the risk of dengue virus transmission in the region.

Materials and methods

Study area

This entomological study was conducted in the subregion of Urabá, specifically in three neighborhoods located in the municipalities of Apartadó and Turbo, which are part of the department of Antioquia in

Colombia. The study was carried out between February 2021 and November 2022, in an area known for endemic dengue transmission.

Apartadó Municipality: The municipality of Apartadó (7º 53' 00'' N; 76º 38' 00'' W) covers an area of 600 km^2 and has a population of 112,396 inhabitants. The population density in Apartadó is 187 inhabitants per km^2, which is higher than the population densities of both the Urabá region (40 inhabitants per km^2) and the Antioquia department (93 inhabitants per km^2) (17). The average temperature in Apartadó is 28°C, and the municipality is situated at an altitude of 25 meters above sea level. Among the neighborhoods in Apartadó, the Serranía neighborhood (7° 53'7.65'' N, 76° 38'2.51'' W) was selected for this study due to its high incidence of dengue cases in recent years. Serranía is characterized as a low to middle-income area with planned urbanization, high population density and basic infrastructure services such as piped water supply, sewerage, and regular garbage collection (Fig 1).

Nueva Colonia is a district located within the municipality of Turbo (7°56'06.9 ''N- 76°43'01.6 ''W) and is situated at an altitude of 2 meters above sea level. It functions as a population center with approximately 19,364 inhabitants. The population density in Nueva Colonia is 72

inhabitants per km^2, with 66.6 % residing in 12 neighborhoods distributed among 3,377 houses, and the remaining 33.4 % residing in 20 villages (18). For this study, the neighborhoods of 29 de noviembre (7° 53'6.95'' N, 76° 38'1.53'' W) and 24 de diciembre (7° 56'1.93'' N, 76° 42'9.87'' W) were selected in Nueva Colonia. These neighborhoods have experienced a significant number of dengue cases and child deaths related to dengue (19) (Fig 1). They are characterized by unplanned urbanization, high population density, and low-income levels. The streets in these neighborhoods are unpaved, and there are puddles due to the lack of sewage systems. Additionally, the population lacks sufficient access to drinking water and regular solid waste collection services.

Fig 1. Map of the study neighborhoods. Serranía neighborhood, Apartadó, and 29 de noviembre and 24 de diciembre neighborhoods in the Nueva Colonia district, Turbo. Prepared using ArcGIS 10.5 (Esri, CA, USA).

Information on probable cases of dengue fever

The information on probable cases of dengue fever was obtained from the National Public Health Surveillance System (SIVIGILA) in Apartadó and Turbo for the period 2021-2022. These cases include both confirmed and suspected cases of dengue, which were identified

based on symptoms consistent with dengue and meeting at least one of the criteria established by the World Health Organization (20,21).

Weather information

Monthly records of precipitation (mm), relative humidity (%), and temperature (°C) for the period 2021-2022 were obtained from two meterological stations of the Institute de Hidrología, Meteorología y estudios ambientales (IDEAM) located in Apartadó, Antioquia, Colombia. These data were used to perform correlation analyses between climatological variables and vector density in the three neighborhoods of the two municipalities. It should be noted that the weather information from the meteorological stations in the municipality of Turbo was not available. However, it is important to mention that the township of Nueva Colonia, Turbo, is geographically closer to the municipality of Apartadó than to the municipality of Turbo.

Sample

With a margin of error of 5 %, a population proportion (P) of 50 %, and a design effect of 1.1, a sample size of 1,608 dwellings was calculated.

Taking into account a potential loss of 10 %, a total of 1,518 dwellings were included in the entomological sampling across the three neighborhoods. Out of these, 90 dwellings (5.6 %) did not provide a response, resulting in a final sample size of 1,428 dwellings.

The inspection of dwellings and the collection of adult mosquitoes were conducted eight times over a two-year period, from February 2021 to November 2022. A sampling interval of every 10th dwelling was used, and randomly generated numbers assigned to each dwelling were used to determine the starting point from which they were visited, following a clockwise direction. The households served as sampling units for the study.

Entomological surveys

The inspection of houses was conducted using a Google® form, which automatically recorded the relevant information in a Microsoft Excel® workbook. The recorded data included the date, house number, owner's identification and name, number of inhabitants per house, types of breeding sites found, their positivity, and the number of mosquitoes collected. The geographical coordinates of each inspected

house were obtained using a GPS (Geographical Positioning System). The entomological surveys followed the operational guide provided by the World Health Organization for assessing the productivity of *Ae. aegypti* breeding sites (22). The survey team consisted of a community leader, two research assistants, and a biologist. The team thoroughly inspected the dwellings, examining all potential water containers for both immature and adult mosquito stages. The intradomiciliary space, which included areas inside the dwelling such as bedrooms, living rooms, bathrooms, and kitchens, was inspected. Additionally, the peridomiciliary space, which encompassed the open areas of the property such as patios, gardens, and terraces, was also examined. A container containing larvae or pupae was considered positive for the presence of immature mosquito stages.

Pupae collection

The pupae found in the breeding sites were collected by using a standard plastic scoop and transferring them to a small container. From there, they were transferred to labeled plastic jars filled with water using a Pasteur pipette. In the laboratory, the collected pupae

were moved to covered pupae emergence containers, where they were left until adult mosquitoes emerged.

For species identification adult mosquitoes were sacrificed by placing them in a -4 °C environment for 30 minutes. Morphological identification of the mosquitoes was carried out using a stereomicroscope at the Entomology laboratory of the Inst[illegible]o Colombiano de Medicina Tropical - CES University (ICMT-CES) in Apartadó, Antioquia, Colombia.

The emerged female *Ae. aegypti* mosquitoes were stored in pools containing 8 to 10 mosquitoes each. The mosquitoes were placed in 1.5 mL cryovial tubes with RNAlater and labeled according to the collection site. The samples were then preserved at 4 °C in the laboratory and later frozen at -80 °C for further processing (23).

Collection of adult mosquitoes

The collection of adult mosquitoes was conducted in the same houses that were inspected. Mosquitoes were captured using Prokopack® aspirators (John W. Hock Co., Gainesville, FL, USA) for a duration of 20 minutes. The collection was carried out between 8:00 AM and 4:00

PM, following the sampling procedure described by Prokopeck et al. (24). After collection, captured mosquitoes were transferred to the Entomology laboratory of the ICMT-CES in Apartadó, Antioquia, Colombia. In the laboratory, the mosquitoes were identified and processed in the same manner as the emerged mosquitoes described previously.

Mosquito processing and DENV detection

The pools of *Ae. aegypti* females were transported to the ICMT-CES laboratory in Medellín, Antioquia, Colombia for further processing. RNA extraction from each pool of mosquitoes was performed using the Quick-RNA™ Tissue/Insect Microprep kit from Zymo Research, following the manufacturer's protocol.

For the detection of dengue virus (DENV), the primers and probe suggested in the protocol by Gurukumar et al. (25) were used. To identify the serotype of the virus, the set of primers and primers described by Santiago et al. (26). were used. All reverse transcription-polymerase chain reaction (RT-PCR) reactions were carried out using the SuperScript® III Platinum® one-step quantitative system. The

reactions were performed on a Rotor-Gene Q thermocycler from QIAGEN®. The thermocycling parameters consisted of reverse transcription at 50 °C for 30 minutes, followed by denaturation at 95 °C for 2 minutes. Amplification and fluorescence detection were performed at 95 °C for 15 seconds and 60 °C for 1 minute, respectively, for a total of 40 cycles. Each probe's amplification curves were evaluated, and a value of Cq ≥ 37 was considered negative for DENV presence.

Data analysis

The data analysis was conducted using Microsoft Excel 2016 (Microsoft Corporation, Washington, USA). The first step involved calculating the proportion of mosquitoes by species based on the collected data. This information was used to determine the prevalence of *Ae. aegypti* and other mosquito species. Additionally, the data on dwellings with water containers were organized and presented in tables. To evaluate the risk of dengue virus (DENV) transmission, various dengue risk indicators were utilized. This included calculating the aedic indices, which provide information on the density of *Ae. aegypti* mosquitoes in relation to the number of houses inspected. The

mosquito density, spatial distribution, and their correlation with climatological variables such as precipitation, temperature, and relative humidity were also examined. Furthermore, the rate of DENV infection was determined to assess the likelihood of viral transmission.

The level of infestation by *Ae. aegypti* mosquitoes and the risk of dengue transmission were assessed using: (i) the housing index (HI), as the percentage of houses infested with larvae and/or pupae; (ii) the deposit index (DI), as the percentage of water reservoirs infested with larvae and / or pupae; and (iii) the Breteau index (BI), as the number of positive reservoirs per 100 houses inspected in a specific location.

Based on WHO criteria, if the BI exceeds 50, HI exceeds 35, and DI exceeds 20, the risk of dengue transmission is considered high. When the BI falls between 5 and 50, it indicates a high density of *Ae. aegypti* mosquitoes and an increased risk of dengue transmission. Conversely, when the BI is below 5, HI is below 4, and DI is below 3, the likelihood of dengue transmission is considered low (27). Additionally, indices based on female *Ae. aegypti* mosquitoes were used to calculate mosquito density for each dwelling.

To assess the positivity of *Ae. aegypti* mosquitoes, the percentage of infested houses with at least one female *Ae. aegypti* mosquito was calculated. The index of female *Ae. aegypti* mosquitoes per dwelling surveyed (Female / House) and per inhabitant per dwelling surveyed (Female / Inhabitant) were calculated. To represent the spatial distribution of *Ae. aegypti* presence and overall density indices in each neighborhood, Kernel density relationships were employed. This technique utilizes Kernel density estimation to generate heat maps that highlight areas with higher concentrations of *Ae. aegypti* mosquitoes, helping to identify hotspots of vector infestation. All collected female mosquitoes were retained for DENV detection. The Minimum Infection Rate (MIR) was then calculated as the number of positive clusters (clusters of mosquitoes with DENV infection) divided by the total number of mosquitoes processed, multiplied by 1000. The MIR provides an estimate of the proportion of mosquitoes infected with DENV (28).

All statistical analyses were conducted using Jamovi version 2.2 (29), with a significance threshold (α) of 0.05. The Friedman test was employed to determine if there were significant differences in the density of *Ae. aegypti* mosquitoes among the neighborhoods studied.

This non-parametric test is used when comparing multiple related samples. To analyze the potential relationship between climatic variables and vector density per neighborhood, both Spearman's and Pearson's correlation coefficients were used. The choice between these two correlation coefficients depended on the distribution of the data (30).

Ethical considerations

The research protocol was submitted to the Institutional Bioethics Committee of the ICMT-CES in Medellín, and it received approval under Act 66 on June 14, 2019. Informed consent was obtained from each participant before conducting the entomological survey. The researchers sought written consent to enter their homes and carry out the entomological survey.

Results

Mosquito breeding sites and *Aedes aegypti* presence

The results of the entomological survey showed that out of the 1,518 homes inspected, 91.9 % (1,394 homes) had water reservoirs. Among the three neighborhoods, the highest proportion of dwellings with water reservoirs was found in the 24 de diciembre neighborhood, at 99.2 %. The neighborhood of 29 de noviembre had a similarly high proportion, with 99.0 %. The Serranía neighborhood had a slightly lower proportion, with 93.7 %.

Out of the 2,307 water reservoirs inspected, 40.5 % (935) were found to have larvae and / or pupae of *Ae. aegypti* mosquitoes. Among the positive reservoirs, pools or low concrete tanks had the highest positivity rate, accounting for 25.6 % (591 / 935) of the total. The 29 de noviembre neighborhood showed a higher infestation rate compared to other neighborhoods, at 18.2 % (421 reservoirs) (Table 1).

Table 1. Type and number of water reservoirs infested with larvae or pupae of *Aedes aegypti*.

Deposit type	Serranía		29 de noviembre		24 de diciembre		Total	
	Positive deposits n (%)	Deposits n	Positive deposits n (%)	Deposits n	Positive Deposits n (%)	Deposits n	Positive deposits Total N (%)	Total deposits N
Low concrete tanks	103 (4.5)	493	257 (11.1)	470	231 (10.0)	396	591 (25.6)	1359
Plastic tanks	22 (0.9)	90	67 (2.9)	370	41 (1.7)	216	130 (5.6)	676
Bins	13 (0.6)	88	41 (1.7)	263	44 (1.9)	298	98 (4.2)	649
Tires	3 (0.1)	5	20 (0.9)	68	10 (0.4)	17	33 (1.4)	90
Others*	24 (1.0)	53	36 (1.6)	141	23 (1.0)	54	83 (3.6)	248
**Total**	**165 (7.1)**	**729**	**421 (18.2)**	**928**	**349 (15.1)**	**650**	**935 (40.5)**	**2307**

* Bottles, disposable cups, pet drinking fountains.

The overall index of *Ae. aegypti* presence in dwellings (HI) was 48.9 % (95 % CI: 39.2 - 58.6 ± 24.2). The highest HI was recorded in the 24 de diciembre neighborhood (68.9 %, 95 % CI: 61.9 - 75.8 ± 10.1). The overall deposit index (DI) was 29.5 % (95 % CI: 25.1 - 34.0 ± 11.1), with the 24 de diciembre neighborhood having a higher DI (36.4 %, 95 % CI: 30.2 - 42.7 ± 9.01) compared to the other two neighborhoods. The overall Breteau index was 70.2 (95 % CI: 53.6 - 86.9 ± 41.6), with the highest value in the 29 de noviembre neighborhood (100.4, 95 % CI: 77.0 - 124.0 ± 33.7) (Fig 2).

Fig 2. Overall housing, deposits and Breteau indices by neighborhood. 2021-2022.

Species composition and density of mosquitoes collected

At the end of entomological sampling in the Serranía neighborhood, Apartadó, and in the neighborhoods 29 de noviembre and 24 de diciembre in Nueva Colonia, Turbo, during the years 2021 and 2022, a total of 20,050 mosquitoes were collected. Of these, 72.8 % (14,618) were *Culex* spp, 27.1 % (5,408) were *Ae. aegypti* (2,564 females and 2,844 males, with a female to male ratio of 0.9:1.1), and 0.12 % (24 individuals) were *Anopheles albimanus*. The highest proportion of *Ae. aegypti* mosquitoes was collected in the 24 de diciembre neighborhood, accounting for 12.3 % (n = 2,462). (Table 2).

Table 2. Number and Percentage of Adult Mosquitoes Collected by Neighborhood and Species.

Neighborhood	Species	Female (n)	Male (n)	Total (N)	%
Serranía	*Aedes aegypti*	555	745	1300	97.4
	Culex spp.	28	3	31	2.3
	Anopheles albimanus	4	0	4	0.3
	Subtotal	587	748	1335	6.7
29 de noviembre	*Aedes aegypti*	816	830	1646	13.1
	Culex spp.	5040	5865	10905	86.8
	Anopheles albimanus	12	4	16	0.1
	Subtotal	5868	6699	12567	62.7

24 de diciembre	*Aedes aegypti*	1193	1269	2462	12.3
	Culex spp.	1777	1905	3682	59.9
	Anopheles albimanus	3	1	4	0.1
	Subtotal	2973	3175	6148	30.7
	**Total**	**9428**	**10622**	**20050**	

Dwellings infested with *Ae. aegypti* adults accounted for 57.7 % (876) of all dwellings, and of which 48.9 % (742) were infested with females and 47.8 % (726) with males. The highest number of *Ae. aegypti* was observed in the 24 de diciembre neighborhood, with 514 mosquitoes captured in February 2022 (Fig 3).

Fig 3. Number of *Ae. aegypti* adults collected in the dwellings of the three study neighborhoods during the 2021-2022 period.

The mean density of *Ae. aegypti* in the three neighborhoods across the eight samplings was 1.74 mosquitoes per dwelling (95 % CI: 1.38 - 2.11 ± 1.29), and more males than females were collected: 2.02 males per dwelling (95 % CI: 1.52 - 2.51 ± 1.23) versus 1.47 females (95 % CI: 0.94 - 2.0 ± 1.32), a significant difference (Friedman's test: $Q = 5.26$, $df = 1$, $p = 0.02$) (Fig 4). Furthermore, there was no variation in the number of female mosquitoes captured per dwelling, as indicated by the coefficient of variation (CV) ≤ 1 (CV = SD/mean = 0.89).

Fig 4. *Aedes aegypti* adults collected per household in the three neighborhoods during the 2021-2022 period. a. Number of *Aedes aegypti* adult females and males collected per household. b. Number of *Aedes aegypti* females collected per household. The box plot shows the median, interquartile range (IQR) and mean (■) values.

The mean density of *Ae. aegypti* females and males per inhabitant in each of the dwellings in the three neighborhoods across the eight samplings was 0.53 (95 % CI: 0.45 - 0.61 ± 0.28). More males than females were collected: 0.55 males / inhabitant (0.43 - 0.66 ± 0.28) compared to 0.51 females / inhabitant (95 % CI: 0.39 - 0.63 ± 0.29). There were no significant differences between males and females in terms of density per inhabitant (Friedman's test: $Q = 1.64$, $df = 1$, $p = 0.20$) (Fig 5).

Fig 5. *Aedes aegypti* adults collected per inhabitant in the three neighborhoods during the 2021-2022 period. a. Number of *Aedes aegypti* adult females and males collected per inhabitant. b. Number of *Aedes aegypti* females collected per inhabitant. The box plot shows median, interquartile range (IQR) and mean (■) values.

Spatial distribution of *Aedes aegypti* density

Figure 6 shows the spatial distribution of *Ae. aegypti* density in the surveyed dwellings during the entire study period (Eight cycles: 2021-2022) with Kernel density ratio maps. The maps highlight hot spots of

the presence of immature stages and females of *Ae. aegypti* in the surveyed neighborhoods, indicating areas of high density.

Fig 6. Spatial distribution according to the presence of immature stages and females of *Ae. aegypti* in dwellings with their respective Kernel density ratio maps in general (eight cycles: 2021-2022). Serranía (A: Presence, B: Females per dwelling, C: Females per inhabitant), 29 de noviembre and 24 de diciembre (D: Presence, E: Females per dwelling, F: Females per inhabitant).

Relationship of climatic variables and vector density

In the Serranía neighborhood, the vector density of *Ae. aegypti* females per inhabitant did not show a significant correlation with temperature (rs = 0.29, p = 0.48), relative humidity (rho = -0.200, p = 0.63), or precipitation (rs = -0.027, p = 0.95). However, in the 29 de noviembre and 24 de diciembre neighborhoods, there were significant correlations observed. In the 29 de noviembre neighborhood, there was a significant negative correlation between vector density and relative humidity (rs = -0.78, p = 0.022) and a significant positive correlation between vector density and precipitation (rs = 0.750, p = 0.033) (29 nov: rs = 0.750, p = 0.033; 24 dic: rs = 0.733, p = 0.038).

Virus detection assays

For the detection of Dengue virus (DENV) infection, a total of 249 groups or pools consisting of 2,131 *Ae. aegypti* females were tested using a one-step RT-PCR assay. Among these pools, three were found to be positive for DENV infection. The positive mosquitoes were collected from the 24 de diciembre neighborhood (2 pools) and the Serranía neighborhood (1 pool). Most of the infected females were found within the households. The minimum infection rate (MIR) for DENV was calculated as 1.4 (3 positive pools / 2,131 total individual mosquitoes processed × 1000), indicating the number of positive pools per 1,000 mosquitoes processed. The percentage of positive pools was calculated as 1.2 % (3 pools / 249 pools).

During this period, 77 cases of dengue fever were recorded, 56 of which were reported by SIVIGILA and 21 captured by the project in the neighborhoods mentioned, being 14, 5 and 5 cases in Serranía, 29 de noviembre 29 and 24 de diciembre 24, respectively. Table 3 describes the number of human cases detected and the positivity ratio, as well as the serotypes found in patient samples and in mosquitoes.

Table 3. Description of dengue virus infection and distribution of serotypes in human patients and mosquitoes, according to neighborhood.

Neighborhood	Human cases notified SIVIGILA	Human cases captured Project	Human positive dengue cases	% human positivity	Patient serotypes	Number of pools analyzed	Number of Positive pools	% positive mosquitoes	Serotype
**Serranía**	43	19	14	73.7	DEN-1	58	1	1.7	DEN-1
**24-dic**	8	10	5	50.0	DEN-1 y DEN-2	102	2	2.0	DEN-1 y DEN-2
**29-nov**	5	4	2	50.0	DEN-1	89	0	0.0	Undetermined
**Total**	**56**	**33**	**21**	**63.6**	**DEN-1 y DEN-2**	**249**	**3**	**1.2**	**DEN-1 y DEN-2**

Discussion

The high aedic indices observed in our study indicate a substantial presence of mosquito breeding sites within the study areas. The high-density rates of *Ae. aegypti* mosquitoes further highlights the potential for increased transmission of dengue fever in these neighborhoods. This observation aligns with previous studies conducted in the Antioquia region, which have reported similar patterns (31–34), for instance, a study conducted in Urabá documented a 32.5 % (32) positivity rate in dwellings, which is lower than the 49 % recorded in this study. It is worth noting that the comparison between our study and previous investigations should be interpreted with caution due to potential variations in study design, sampling methods, and

geographical factors. However, the consistently high aedic indices across different studies in the Antioquia region suggest a persistent and concerning issue of mosquito-borne disease transmission.

The high density of *Ae. aegypti* observed in this study is likely attributed to several factors, including the presence of numerous water reservoirs and the abundance of immature mosquitoes indoors. The shortage of water in the area may have prompted households to store water in tanks within their homes, leading to a higher number of breeding sites. Improper waste disposal and the prevalence of plastic tanks, buckets, and other water storage containers also contribute to the availability of suitable breeding sites for mosquitoes.

It is noteworthy that a significant proportion of water containers were found to harbor mosquito larvae and were left uncovered. This highlights the need for targeted mosquito control measures that prioritize breeding habitats, particularly focusing on water reservoirs suitable for long-term storage.

The overall housing, deposit, and Breteau indices observed in our study were lower than those reported by Alarcón et al. in 2014 in the Urabá region, specifically in one neighborhood of the municipality of

Apartadó. Alarcón et al. documented values of 58.82 %, 58.82 %, and 111.8 for housing, deposits, and Breteau indices, respectively (31), These differences may be attributed to variations in sampling methods, climatic conditions, and local contexts between the two studies. Comparing the results to another study conducted in the El Manzanillo, Itagüí district in Antioquia, the housing and deposit indices were found to be similar, with values of 48.5 % and 21.3 %, respectively (34). However, the Breteau index in that study was lower at 33. These findings suggest some consistency in housing and deposit indices between the studies, while the variation in the Breteau index may be attributed to differences in mosquito breeding habitats and environmental factors.

This study highlights the elevated aedic indices in the Nueva Colonia neighborhood, suggesting a higher risk of dengue virus transmission for its residents. The comparatively higher indices in the 24 de diciembre and 29 de noviembre neighborhoods, in contrast to the Serranía neighborhood, can be attributed to various factors. These neighborhoods, characterized by lower socioeconomic status, may have intra-domiciliary and peridomiciliary environments with inadequate water storage practices, such as low tanks, plastic

containers, and uncovered garbage cans, which provide favorable breeding sites for mosquitoes.

These observations emphasize the importance of implementing targeted mosquito control measures in these neighborhoods to mitigate the risk of DENV transmission. Effective control practices may include regular inspection and elimination of potential breeding sites, promoting proper water storage practices, and raising awareness among residents about the importance of maintaining clean and mosquito-free environments.

The relationship between human density and vector density, particularly the number of *Ae. aegypti* females, is an important indicator of the risk of dengue transmission, as highlighted by previous studies (35). This human-vector proximity has been reported by different authors. For instance, a study conducted in Taiwan in 1994 reported a density of 0.07 females / inhabitant (36), while studies in Colombia described densities of 0.5 females / inhabitant (37) and 2.1 females / dwelling (30). In other studies conducted in Ibagué, Colombia, Manta, Ecuador, and Posadas, Argentina, densities of 2.2, 1.13, and 1.80 females / dwelling, respectively, were observed, with an average of 1.71 females / dwelling across the three countries (38). Similarly, a

study conducted in Brazil reported densities of 1.60 females / dwelling and 0.42 females / inhabitant (35). The present study found densities of 1.47 females / dwelling and 0.51 females / inhabitant, with the majority of female *Ae. aegypti* mosquitoes collected in the Nueva Colonia neighborhood, particularly in the 24 de diciembre neighborhood. These findings align with the results of previous studies conducted in Latin American countries, suggesting a comparable risk of dengue transmission in these areas.

The findings of this study indicate that the vector density, specifically the number of *Ae. aegypti* females per inhabitant, in the Serranía neighborhood is not significantly correlated with the environmental variables of temperature, humidity, or precipitation. This suggests that factors other than these climatic variables may be influencing the vector density in this particular neighborhood. On the other hand, the neighborhoods of 29 de noviembre and 24 de diciembre show a significant correlation between vector density and relative humidity as well as precipitation. This implies that the abundance of *Ae. aegypti* females in these neighborhoods is influenced by the levels of humidity and rainfall. These environmental conditions may create suitable breeding habitats for mosquitoes, resulting in increased vector density.

Additionally, when considering the number of females per dwelling, only the neighborhood of 24 de diciembre shows a significant correlation with precipitation. This neighborhood, which also recorded the highest number of dengue cases among the two neighborhoods studied in the Nueva Colonia locality, may be more susceptible to dengue transmission due to the combination of high vector density and favorable environmental conditions.

Being located near the equator, as suggested by Carrasquilla et al., (30), Colombia experiences relatively stable temperatures throughout the year, which may explain the lack of significant correlation between temperature and vector density in the Serranía neighborhood. Nevertheless, it is important to note that numerous studies have demonstrated the influence of climatic variables on dengue transmission. Factors such as temperature, humidity, and precipitation have been identified as key drivers of mosquito population dynamics and dengue transmission patterns in various regions (39).

The identification of hotspot foci with a specific pattern in the spatial distribution of immature and adult female *Ae. aegypti* aligns with previous research highlighting the focal nature of *Ae. aegypti* distribution. It is widely observed that the distribution of *Ae. aegypti*

mosquitoes tends to be highly localized, forming clusters or hotspots of high vector abundance within small clusters of houses.

These hotspots of high vector abundance may exhibit temporal instability, meaning they can vary in location and intensity over time (40,41).

The findings of this study suggest that the use of indicators of female density per house and per inhabitant provides a more accurate representation of the spatial distribution of *Ae. aegypti* compared to using presence / absence data of immature stages and females. This is in contrast to a recent study conducted in Brazil, where no consistent pattern in the spatial distribution of *Ae. aegypti* eggs, larvae, or adults was observed using separate entomological indicators (42). The example from Apartadó and Carepa, where hotspots of *Ae. aegypti* infestation were identified based on egg density, highlights the utility of using specific entomological indicators for targeted vector control activities (31). Similarly, in Medellín, spatial analysis techniques such as Kernel density and hot spot analysis were employed to identify areas with a higher presence of the vector, aiding in the implementation of effective vector control strategies (43).

The laboratory analysis conducted in this study revealed the presence of DENV serotypes 1 and 2 in mosquitoes collected from the 24 de diciembre and Serranía neighborhoods. This finding is consistent with previous entomo-virological studies conducted in Colombia, where all four DENV serotypes have been detected, with serotypes 1 and 2 being the most predominant (7,28,30,44). Although the infection rates observed in this study were relatively low, with a minimum infection rate of 1.4 and a positivity rate of 1.2 %, the high density and large number of infested water reservoirs indicate a significant risk of DENV transmission in the study areas. It is worth noting that no studies investigating vector infection in the Urabá region were found in the literature review conducted. The presence of DENV infection in mosquitoes can be influenced by various factors, including rainfall, the number of mosquitoes tested, and the circulation of DENV among the human population during the study period. Despite the low number of laboratory-confirmed dengue cases among residents of the study neighborhoods, the detection of DENV serotypes 1 and 2 in both human samples and mosquito pools in the 24 de diciembre neighborhood, as well as the presence of DENV serotype 1 in both

humans and adult mosquito pools in the 24 de diciembre and Serranía neighborhoods, is noteworthy.

Recent studies conducted in Medellín, Antioquia, analyzed pools of *Aedes aegypti* mosquitoes and found a 13 % positivity rate for DENV (45), however there is a lack of studies investigating DENV positivity in mosquitoes in Urabá.

These findings underscore the importance of entomo-virological surveillance as an early warning activity for vector control in endemic areas such as Urabá. Detecting DENV infection in mosquitoes provides valuable information for understanding the transmission dynamics and implementing targeted vector control measures to mitigate the risk of dengue transmission.

Conclusions

This study highlights several important findings regarding the risk of dengue transmission and the presence of *Ae. aegypti* mosquitoes in the neighborhoods under study. The high infestation rates of water reservoirs with mosquito larvae and pupae indicate a conducive environment for mosquito breeding. The presence and density of *Ae.*

aegypti mosquitoes, particularly in the Nueva Colonia neighborhood, suggest an increased risk of DENV transmission in these areas.

The detection of females carrying DENV serotypes 1 and 2 in the study neighborhoods, along with the correlation with dengue cases among residents, further supports the likelihood of local transmission. This emphasizes the importance of targeted vector control measures to reduce the risk of dengue transmission in these communities.

The use of entomological indicators, specifically the presence and density of adult female mosquitoes, provides valuable complementary information to traditional aedic indices. Integrating these indicators into vector surveillance efforts can enhance the understanding of dengue and other arbovirus transmission dynamics, enabling more effective monitoring and control strategies.

Limitations of the study

The study was conducted in only three neighborhoods in two municipalities of Urabá, which may limit the generalizability of the findings to other areas within the region. These neighborhoods were selected based on their high dengue burden and specific urban and

demographic characteristics, which may differ from other municipalities in Urabá.

The study's timeframe and duration may also be considered a limitation. The data collection period and the number of monitoring cycles may not capture the full seasonal dynamics and fluctuations in vector density and viral activity, potentially leading to an incomplete representation of the transmission patterns.

Acknowledgments

The authors would like to extend their heartfelt thanks to the residents who generously granted access to their homes for the purpose of entomological sampling. The authors also acknowledge the invaluable support and assistance provided by the Health *Secretaries* of Apartadó and Turbo in Antioquia, Colombia.

Financing

This work was financially supported by the Ministry of Science, Technology, and Innovation of Colombia under project code 67882.

Conflict of interest

All authors involved in this study declare that there is no conflict of interest.

Authors' contributions

The authors' contributions to the study are as follows:

- **Conceptualization:** Wilber Gómez-Vargas, Margarita Arboleda, Paola Astrid Ríos-Tapias, Katerine Marín-Velásquez.
- **Formal analysis:** Wilber Gómez-Vargas, Margarita Arboleda, Erika Giraldo-Gallo, Angela Segura-Cardona.
- **Acquisition of funds:** Margarita Arboleda, Paola Astrid Ríos-Tapias.
- **Research:** Wilber Gómez-Vargas, Paola Astrid Ríos-Tapias, Katerine Marín-Velásquez.
- **Methodology:** Wilber Gómez-Vargas, Paola Ríos-Tapias, Katerine Marín-Velásquez.

- **Writing - original draft:** Wilber Gómez-Vargas, Margarita Arboleda, Paola Astrid Ríos-Tapias, Katerine Marín-Velásquez.
- **Writing - proofreading and editing:** Wilber Gómez-Vargas, Paola Astrid Ríos-Tapias, Katerine Marin-Velásquez, Erika Giraldo-Gallo, Angela Segura-Cardona, Margarita Arboleda.

References

1. Instituto Nacional de Salud. Eventos por arbovirus [Internet]. Boletín Epidemiológico Semanal; 2022. Available from: https://www.ins.gov.co/buscador-eventos/BoletinEpidemiologico/2022_Bolet%C3%ADn_epidemiologico_semana_33.pdf
2. Ministerio de la Protección Social de Colombia, Instituto Nacional de Salud, Organización Panamericana de la Salud. Gestión para la vigilancia entomológica y control de la transmisión de dengue. Bogotá: MPS; 2012.
3. Olano VA. Editorial: *Aedes aegypti* en el área rural: implicaciones en salud pública. Biomédica. 2016;36(2):169-73. doi:10.7705/biomedica.v36i2.3374
4. Stewart Ibarra AM, Luzadis VA, Borbor Cordova MJ, Silva M, Ordoñez T, Beltrán Ayala E, et al. A social-ecological analysis of community perceptions of dengue fever and *Aedes aegypti* in Machala, Ecuador. BMC Public Health. 2014;14(1):1135. doi:10.1186/1471-2458-14-1135
5. Méndez F, Barreto M, Arias JF, Rengifo G, Muñoz J, Burbano ME, et al. Human and mosquito infections by dengue viruses during and after epidemics in a dengue–endemic region of Colombia. Am. J.

Trop. Med. Hyg. 2006;74(4):678–83. doi:10.4269/ajtmh.2006.74.678

6. Gómez-Palacio A, Suaza-Vasco J, Castaño S, Triana O, Uribe S. Aedes albopictus (Skuse, 1894) infected with the American-Asian genotype of dengue type 2 virus in Medellín suggests its possible role as vector of dengue fever in Colombia. Biomédica. 2017;37(Supl.2):135-42. doi:10.7705/biomedica.v37i0.3474

7. Pérez-Pérez J, Sanabria WH, Restrepo C, Rojo R, Henao E, Triana O, et al. Vigilancia virológica de *Aedes (Stegomyia) aegypti* y *Aedes (Stegomyia) albopictus* como apoyo para la adopción de decisiones en el control del dengue en Medellín. Biomédica. 2017;37(Supl.2):155-66. doi:10.7705/biomedica.v37i0.3467

8. Cámara de Comercio de Medellín para Antioquia. Perfil socioeconómico de la subregión del Urabá [Internet]. Medellín (Colombia); 2015. Available from: https://www.camaramedellin.com.co/Portals/0/Biblioteca/Estudios-economicos/cadenas-productivas-regionales/20-3Perfil%20Uraba_Oct14.pdf?ver=2019-03-01-095036-403

9. Ríos Cadavid JF. Aspectos entomológicos del dengue. Infectio. 2004;8(3):231–5.

10. Barrera R. Recomendaciones para el monitoreo de Aedes aegypti. Biomédica. 2016;36(3):454–62. doi:10.7705/biomedica.v36i3.2892

11. Nascimento KLC, Silva JFM da, Zequi JAC, Lopes J. Comparison Between Larval Survey Index and Positive Ovitrap Index in the Evaluation of Populations of *Aedes* (*Stegomyia*) *aegypti* (Linnaeus, 1762) North of Paraná, Brazil. Environ Health Insights. 2020;14:1–8. doi:10.1177/1178630219886570

12. Focks DA. A Review of Entomological Sampling Methods and Indicators for Dengue Vectors. Gainesville, Florida: TDR/WHO; 2003. 40 p.

13. Rodriguez-Figueroa L, Rigau-Perez JG, Suarez EL, Reiter P. Risk factors for dengue infection during an outbreak in Yanes, Puerto Rico in 1991. Am. J. Trop. Med. Hyg. 1995;52(6):496–502. doi:10.4269/ajtmh.1995.52.496

14. Reiter PJ. Surveillance and control of urban dengue vectors. In: Gubler DJ, Ooi EE, Vasudevan S, Farrar J, editors. Dengue and Dengue Hemorrhagic Fever. Boston, USA; 2014. p. 481–518.

15. Soares ENL, Santos MAB, Macedo LOD, Santos CVB, Agra MCR, Alves LC, et al. Spatial distribution of *Aedes aegypti* (Diptera: Culicidae) in vulnerable areas for the transmission of arboviruses. Rev Soc Bras Med Trop. 2019;52:e20180341. doi:10.1590/0037-8682-0341-2018

16. Arroyave E, Londoño AF, Quintero JC, Agudelo-Florez P, Arboleda M, Díaz FJ, et al. Etiología y caracterización epidemiológica del síndrome febril no malárico en tres municipios del Urabá antioqueño, Colombia. Biomédica. 2012;33(Supl.1):99–107. doi:10.7705/biomedica.v33i0.734

17. DANE. Censo Nacional de Población y Vivienda [Internet]. 2018. Available from: https://www.dane.gov.co/index.php/estadisticas-por-tema/demografia-y-poblacion/censo-nacional-de-poblacion-y-vivenda-2018/informacion-tecnica

18. Gobernación de Antioquia. Corregimiento de Nueva Colonia [Internet]. 2023 [cited 2023 Apr 5]. Available from: https://corregimientos.antioquia.gov.co/corregimiento-nueva-colonia/

19. Alcaldía distrital de Turbo. Información sobre el municipio de Turbo [Internet]. 2020 [cited 2020 Oct 10]. Available from: http://www.turbo-antioquia.gov.co

20. OMS. Dengue: guías para la atención de enfermos en la región de las Américas. 2a ed. Washington, USA.: OPS; 2015.

21. Instituto Nacional de Salud. Protocolo de Vigilancia en Salud Pública de Dengue [Internet]. [cited 2023 May 8]. Available from: https://www.ins.gov.co/buscador-eventos/Lineamientos/Pro_Dengue.pdf

22. WHO. Operational guide for assessing the productivity of Aedes aegypti breeding sites. [Internet]. WHO, World Health Organization; 2011. Available from: https://www.who.int/tdr/publications/tdr-research-publications/soppupal-surveys/en/

23. Reinert JF, Harbach RE, Kitching IJ. Phylogeny and classification of Aedini (Diptera: Culicidae), based on morphological characters of all life stages. Zool J Linn Soc. 2004;142(3):289–368. doi:10.1111/j.1096-3642.2004.00144.x

24. Vazquez-Prokopec GM, Galvin WA, Kelly R, Kitron U. A New, Cost-Effective, Battery-Powered Aspirator for Adult Mosquito Collections. J Med Entomol. 2009;46(6):1256–9. doi:10.1603/033.046.0602

25. Gurukumar K, Priyadarshini D, Patil J, Bhagat A, Singh A, Shah P, et al. Development of real time PCR for detection and quantitation of Dengue Viruses. Virol J. 2009;6(1):10. doi:10.1186/1743-422X-6-10

26. Santiago GA, Vergne E, Quiles Y, Cosme J, Vazquez J, Medina JF, et al. Analytical and Clinical Performance of the CDC Real Time RT-PCR Assay for Detection and Typing of Dengue Virus. PLoS Negl Trop Dis. 2013;7(7):e2311. doi:10.1371/journal.pntd.0002311

27. WHO. Technical guide for a system of yellow fever surveillance. WHO; 1971.

28. Pérez-Castro R, Castellanos JE, Olano VA, Matiz MI, Jaramillo JF, Vargas SL, et al. Detection of all four dengue serotypes in *Aedes aegypti* female mosquitoes collected in a rural area in Colombia. Mem Inst Oswaldo Cruz. 2016;111(4):233–40. doi:10.1590/0074-02760150363

29. The Jamovi Project. Jamovi [Internet]. 2022. Available from: https://www.jamovi.org/

30. Carrasquilla MCC, Ortiz MI, León C, Rondón S, Kulkarni MA, Talbot B, et al. Entomological characterization of Aedes mosquitoes and arbovirus detection in Ibagué, a Colombian city with co-circulation of Zika, dengue and chikungunya viruses. Parasites Vectors. 2021;14(1):446. doi:10.1186/s13071-021-04908-x

31. Alarcón EP, Segura ÁM, Rua-Uribe G, Parra-Henao G. Evaluación de ovitrampas para vigilancia y control de *Aedes aegypti* en dos centros urbanos del Urabá antioqueño. Biomédica. 2014;34(3):409–24. doi:10.7705/biomedica.v34i3.2134

32. López-A. YL, Wolff-E. MI, Escobar-V. JP, Osorio-Q. L. Distribución espacial y habitats larvarios del *Aedes aegypti* en el departamento de Antioquia. 1990. Rev Colomb Entomol. 1992;18(2):63–72.

33. López YL. Vigilancia entomológica de *Aedes aegypti* en el departamento de Antioquia en 1995. Bol Vect. 1995;7:4–17.

34. Giraldo-Hurtado TM, Álvarez-Betancur JP, Parra-Henao G. Factores asociados a la infestación domiciliaria por *Aedes aegypti* en el corregimiento el Manzanillo, municipio de Itagüí (Antioquia) año 2015. Rev Fac Nac Salud Pública. 2018;36(1):34–44. doi:10.17533/udea.rfnsp.v36n1a05

35. Rodrigues MDM, Marques GRAM, Serpa LLN, Arduino MDB, Voltolini JC, Barbosa GL, et al. Density of *Aedes aegypti* and *Aedes albopictus* and its association with number of residents and meteorological variables in the home environment of dengue endemic area, São Paulo, Brazil. Parasites Vectors. 2015;8:115. doi:10.1186/s13071-015-0703-y

36. Chen YR, Hwang JS, Guo YJ. Ecology and control of dengue vector mosquitoes in Taiwan. Kaohsiung J Med Sci. 1994;10:S78–87.

37. Romero-Vivas CME, Falconar AKI. Investigation of relationships between *Aedes aegypti* egg, larvae, pupae, and adult density indices where their main breeding sites were located indoors. J. Am. Mosq. Control Assoc. 2005;21(1):15–21. doi:10.2987/8756-971X(2005)21[15:IORBAA]2.0.CO;2

38. Talbot B, Sander B, Cevallos V, González C, Benítez D, Carissimo C, et al. Determinants of Aedes mosquito density as an indicator of arbovirus transmission risk in three sites affected by co-circulation of globally spreading arboviruses in Colombia, Ecuador and Argentina. Parasites Vectors. 2021;14(1):482. doi:10.1186/s13071-021-04984-z

39. Dibo MR, Chierotti AP, Ferrari MS, Mendonça AL, Chiaravalloti Neto F. Study of the relationship between *Aedes (Stegomyia) aegypti* egg and adult densities, dengue fever and climate in Mirassol, state of São Paulo, Brazil. Mem Inst Oswaldo Cruz. 2008;103(6):554–60. doi:10.1590/S0074-02762008000600008

40. LaCon G, Morrison AC, Astete H, Stoddard ST, Paz-Soldan VA, Elder JP, et al. Shifting Patterns of Aedes aegypti Fine Scale Spatial Clustering in Iquitos, Peru. PLoS Negl Trop Dis. 2014;8(8):e3038. doi:10.1371/journal.pntd.0003038

41. Albrieu-Llinás G, Espinosa MO, Quaglia A, Abril M, Scavuzzo CM. Urban environmental clustering to assess the spatial dynamics of *Aedes aegypti* breeding sites. Geospat Health. 2018;13:654. doi:10.4081/gh.2018.654

42. Souza RL, Nazare RJ, Argibay HD, Pellizzaro M, Anjos RO, Portilho MM, et al. Density of *Aedes aegypti* (Diptera: Culicidae) in a low-income Brazilian urban community where dengue, Zika, and chikngunya viruses co-circulate. Parasites Vectors. 2023;16(1):159. doi:10.1186/s13071-023-05766-5

43. Calle Echeverri DA, Carabali M, Henao E, Henao GP, González S, Restrepo BN. Dinámica espacio-temporal del dengue en la ciudad de Medellín, Colombia. 2008-2013. 2016.

44. Velandia-Romero ML, Olano VA, Coronel-Ruiz C, Cabezas L, Calderón-Peláez MA, Castellanos JE, et al. Dengue virus detection in *Aedes aegypti* larvae and pupae collected in rural areas of Anapoima, Cundinamarca, Colombia. Biomédica. 2017;37(Supl.2):193-200. doi:10.7705/biomedica.v37i0.3584

45. Rúa-Uribe GL, Giraldo-Jaramillo TM, Triana-Chávez O, Rojo R, Henao E, Pérez-Pérez J. Transmisión vertical de virus dengue en *Aedes* spp. (Diptera: Culicidae) en Medellín, Colombia. Rev Colomb Entomol. 2020;46(1):e6973. doi:10.25100/ socolen.v46i1.6973.

Figure

Click here to access/download;Figure;Fig 1. Map of the Study neighborhoods.tif

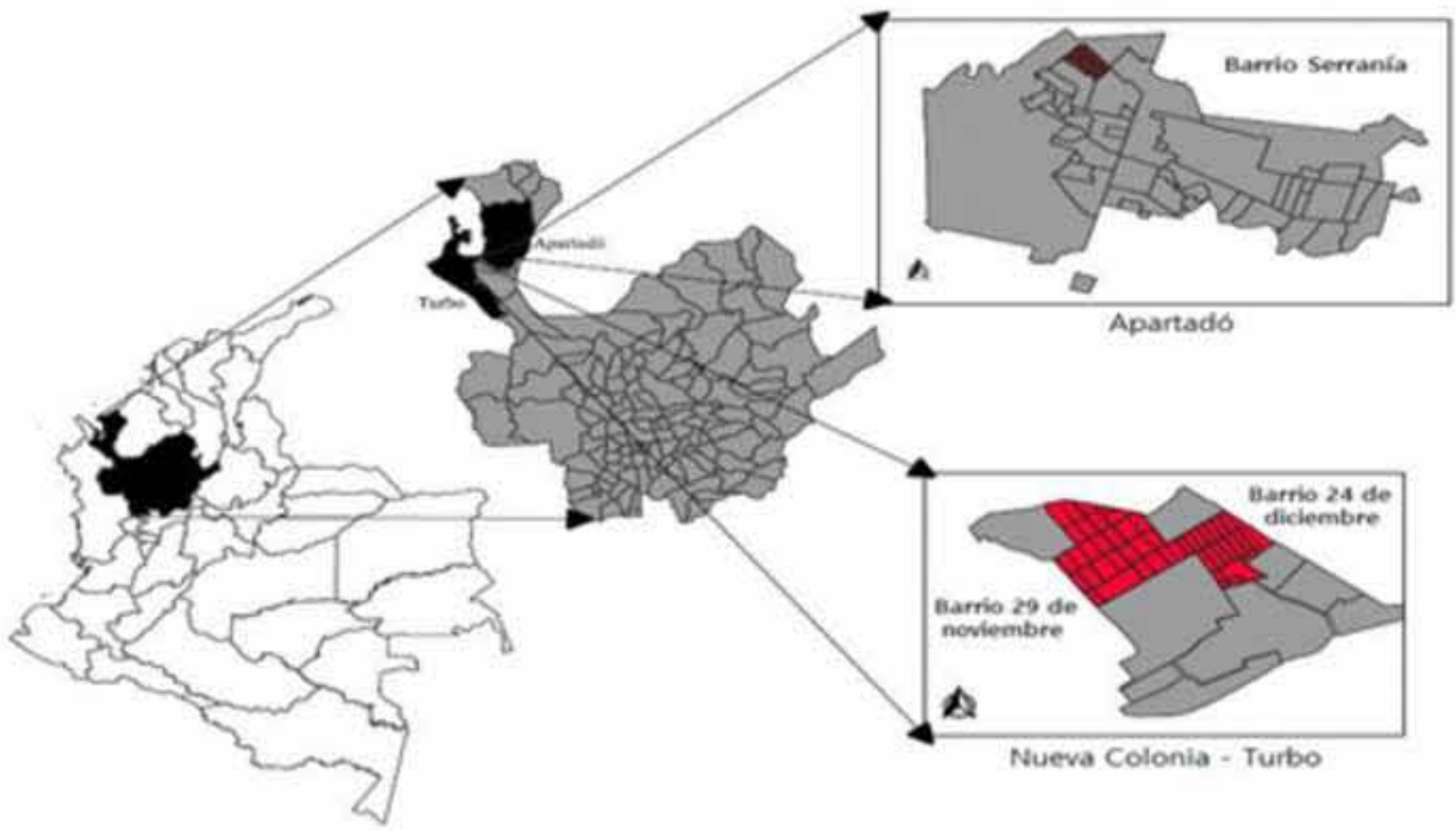

Click here to access/download;Figure;Fig 2. Aedic indices.tif

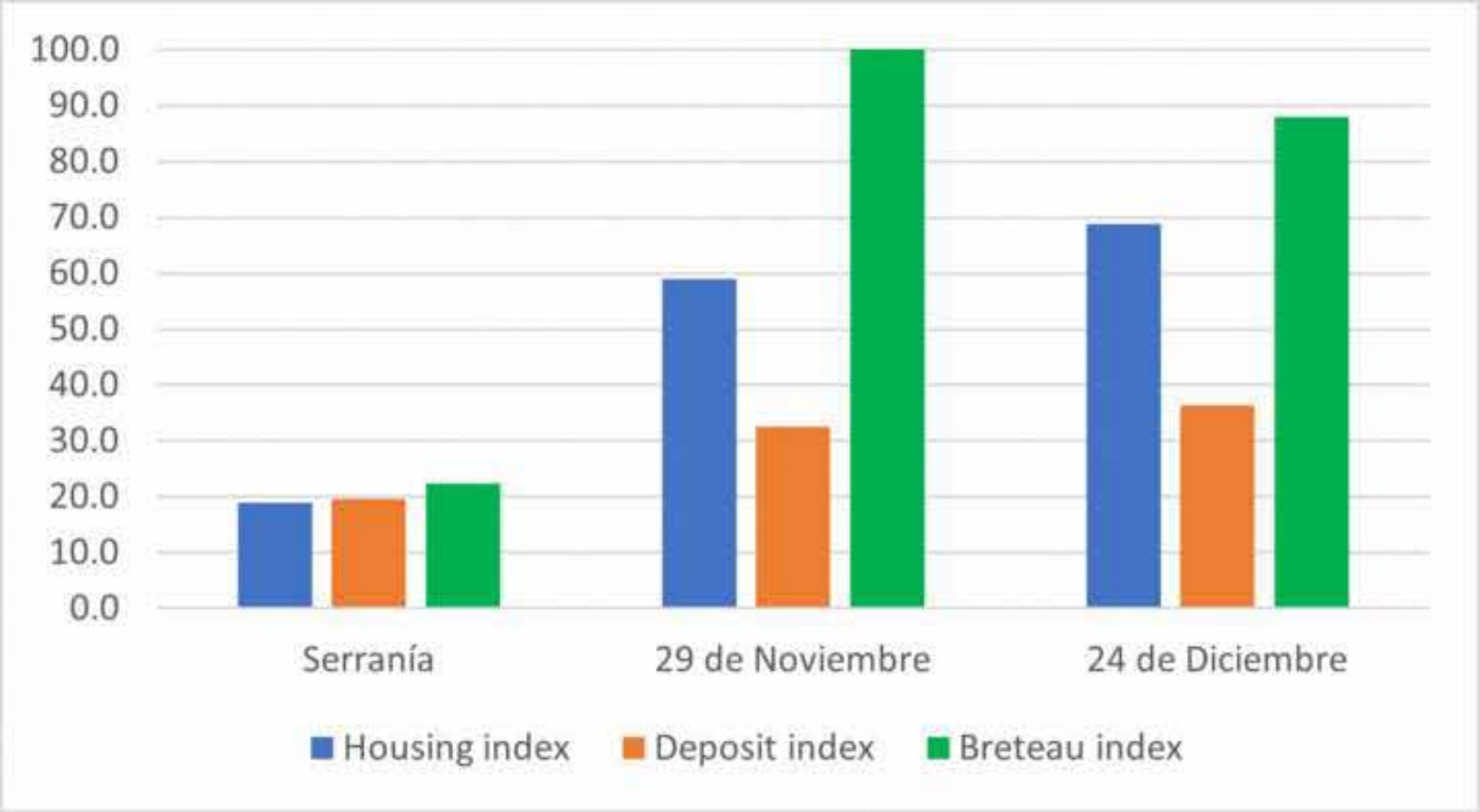

 Click here to access/download;Figure;Fig 3. Number of Ae. aegypti collected.tif

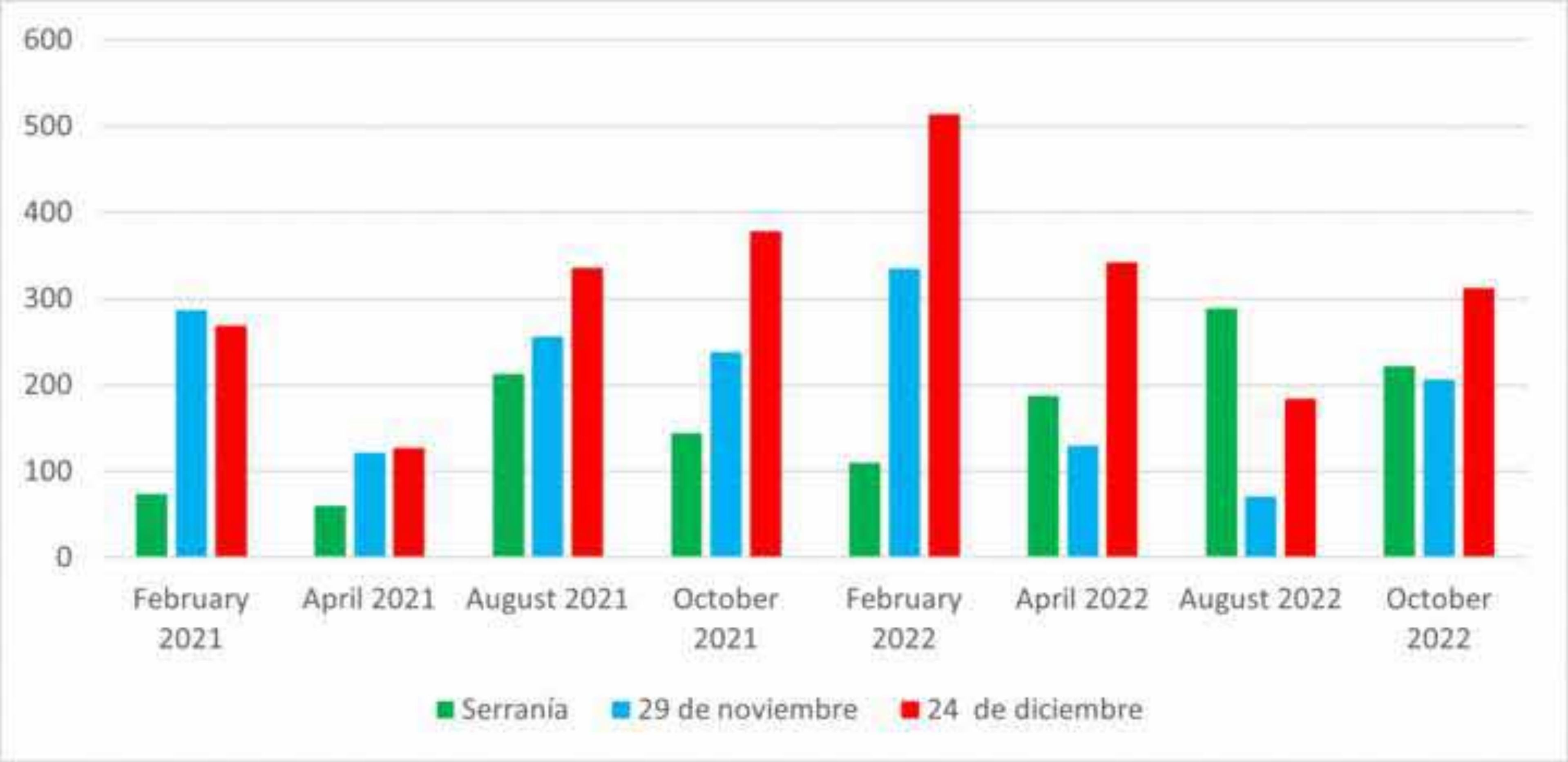

Figure

Click here to access/download;Figure;Fig 4. Number of Ae. aegypti per household.tif

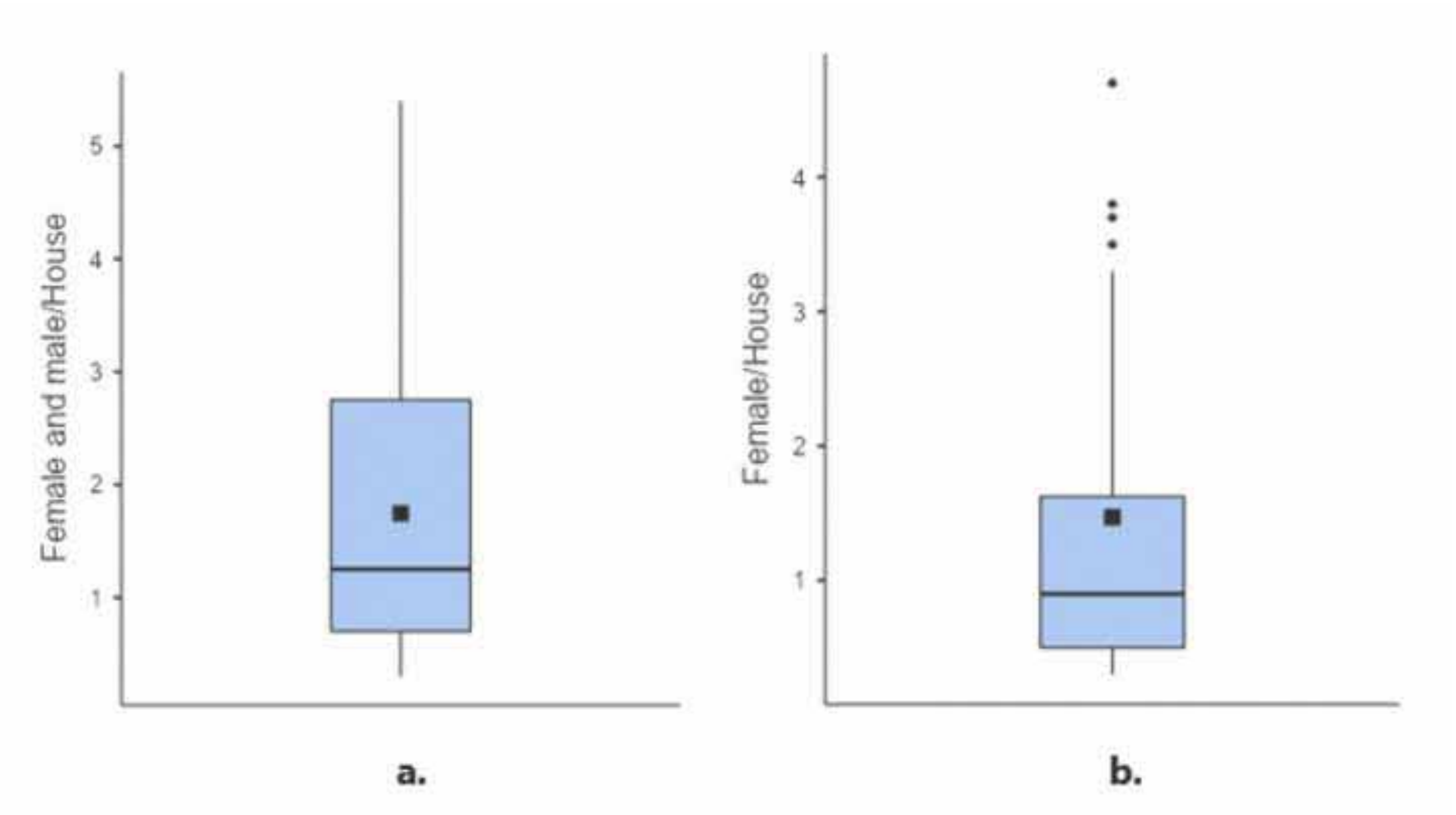

Figure

Click here to access/download;Figure;Fig 5. Number of Ae. aegypti per inhabitant.tif

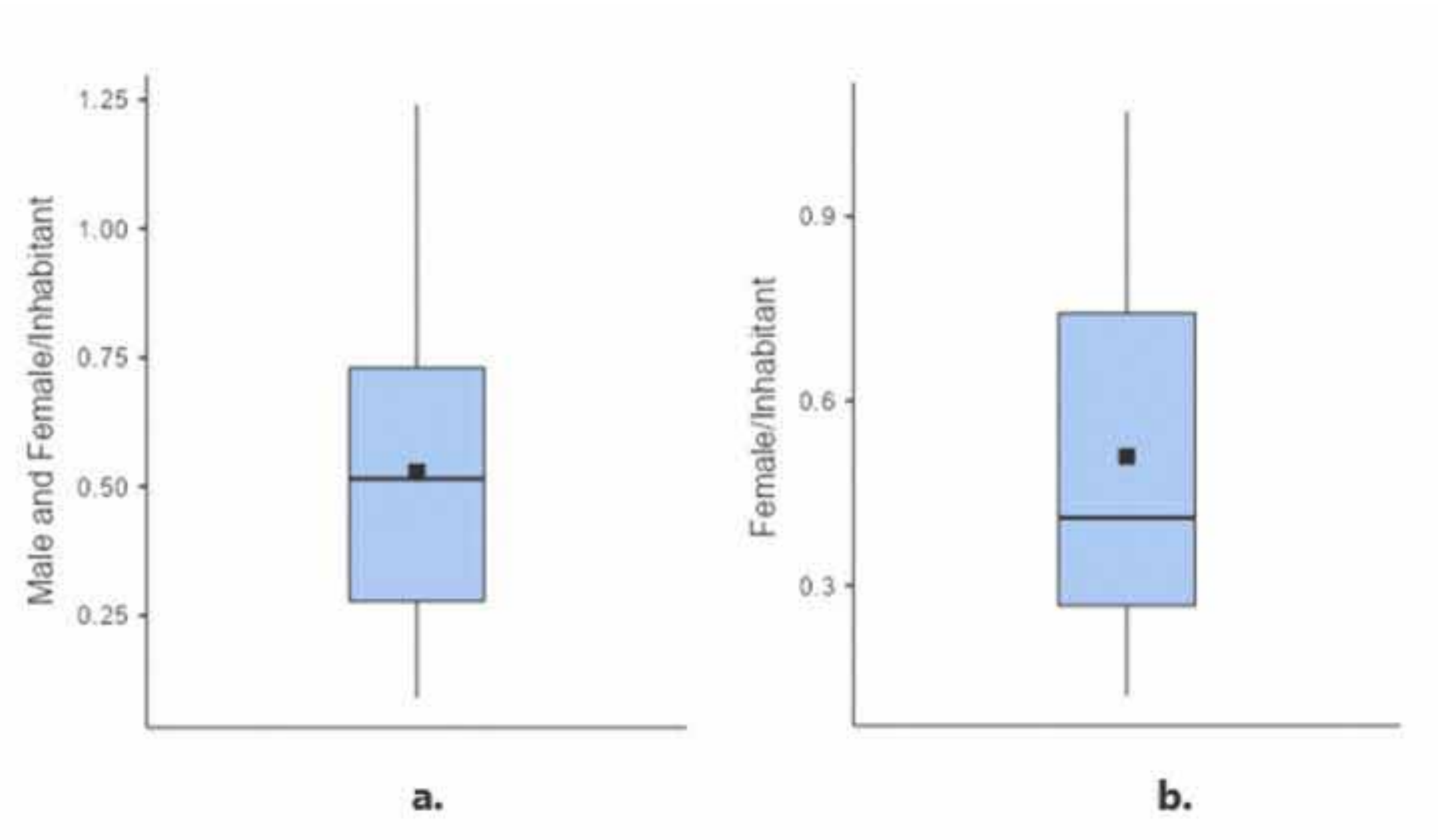

Figure

Click here to access/download;Figure;Fig 6. Spatial distribution of Ae. aegypti.tif

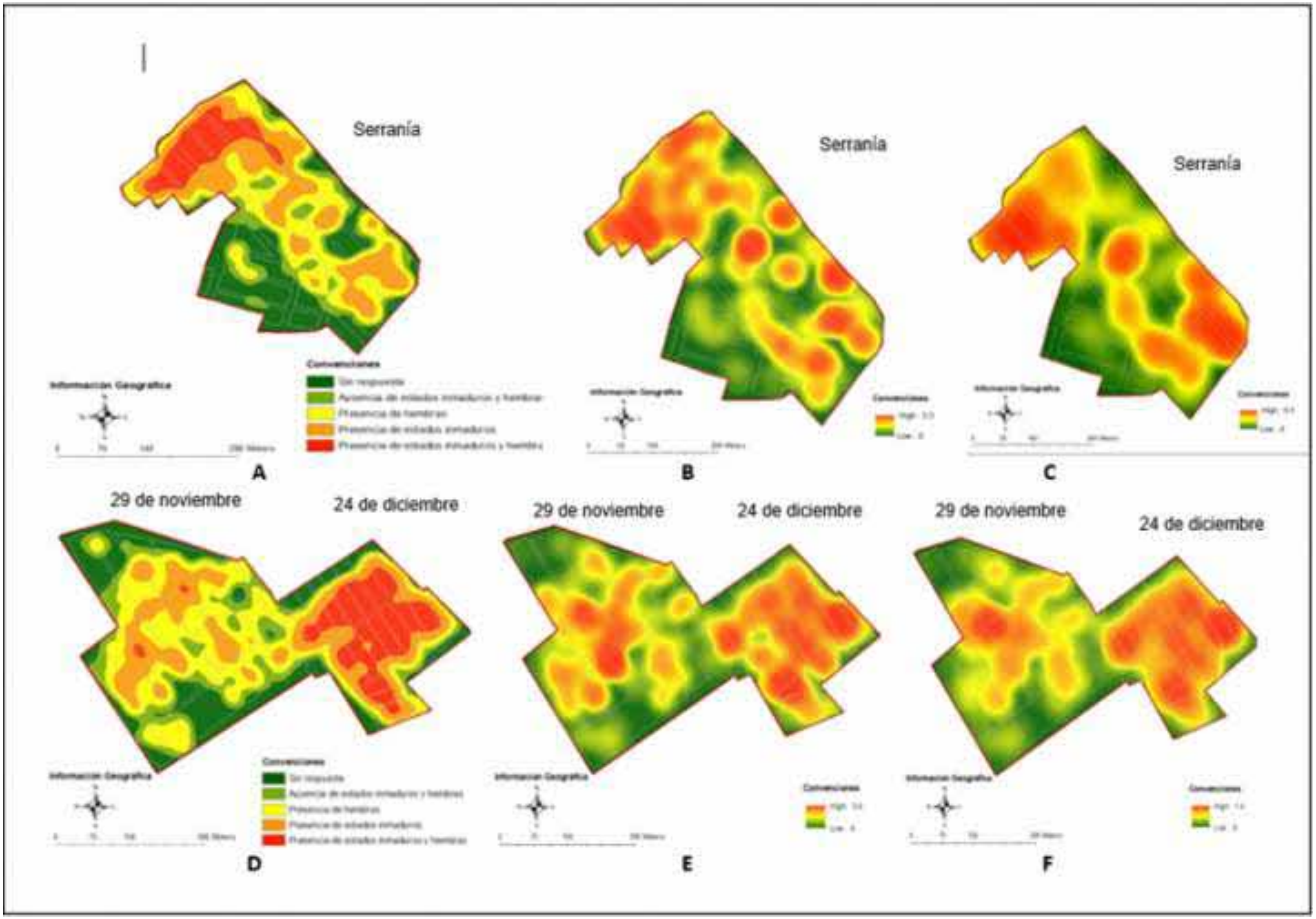

Click here to access/download
Supporting Information
S1. Appendix.xlsx
