# Supplementary material for: Density of Aedes aegypti and dengue virus transmission risk in two municipalities of Northwestern Antioquia, Colombia
Source: PLoS One. 2024 Jan 25;19(1):e0295317. doi: 10.1371/journal.pone.0295317 (PMC10810462; doi:10.1371/journal.pone.0295317)
Supplement: Attachment — Submitted filename: Response to reviewers.docx [file pone.0295317.s003.docx]

Medellín, Colombia, October 18, 2023

Harapan Harapan, MD, PhD

Academic Editor

PLOS ONE

Subject: Response to comments done to the manuscript: Density of *Aedes aegypti* and Dengue Virus Transmission Risk in Two Municipalities of Northwestern Antioquia, Colombia.

**Journal requirements**

1. PLOS ONE style requirements were met, including those related to file naming.
2. Figures 1 and 6 **do not contain** [map/satellite] images that may be copyrighted.

In relation to figure 1, in the text on lines 151 to 154 it is explained that: “This figure was constructed using Arc-Gis software and the area of the neighborhoods was delimited using the maps of Apartadó and Turbo, Antioquia, Colombia”.

In relation to figure 6, in the text on lines 330 to 333 it is explained that: “For the construction of these maps (Figure 6), the traditional Aedes indices and the vector density indices obtained by the project with ArcGIS 10.5 software were used (see supplementary information S1).”.

**Response to reviewer's comments 1**

1. *Suggest to use Aedes instead of aedic throughout the manuscript:* Suggested changes were made throughout the manuscript.

2. *80% - Please check throughout the text*: Suggested changes were made throughout the manuscript.
3. *Please state the time or duration of this occurrence*:

“The Urabá region has been experiencing a predominance of acute febrile syndrome, and dengue infection has been identified as a significant contributor to this syndrome since 2007 (15,16).” (Lines in the text of 146 to 148).

4. *Any citations or data to support this statement?* A quote and data were included that supports this statement. “…was selected for this study due to high number of dengue cases in recent years (2018 to 2020: 82 cases) (18).”. (Lines in the text of 146 to 148).
5. *Positivity for immatures?*: the positivity for immatures states (larvae and pupae). (Lines in the text of 223 to 224).
6. *Please state which taxonomic keys were used for the identification:* was used the key of Rueda (2004). (Line 250).
7. *Suggest to add the number of human cases reported for each period in the secondary axis of Fig 3 to demonstrate the correlation between the mosquito abundance and human cases in the study areas*: The number of human cases reported for each period were added on the X axis in Fig 3.
8. *Is this DEN-1 and DEN-2?:* The table was corrected.
9. *Please discuss these contrasting findings on climatic variable in a broader context based on what has previously been recorded in literature, particularly in the Americas.*

“..which has a good water supply all year round”. (Lines in the text of 580 to 581).

*“…*such as the presence of the vector outside the homes such as disposable containers, tires, among others”. (Lines in the text of 583 to 584).

“In these neighborhoods, due to the poor water supply, the inhabitants are forced to store water throughout the year, especially in dry weather”. (Lines in the text of 590 to 591).

“That is why the rains favor the increase of breeding sites and cases of dengue. In dry periods and low temperatures, vectors decrease, a situation that does not stop the transmission of the disease, due to the active biting behavior of the vector that favors the spread of the virus (41). It can be observed that the impact of rainfall varied according to the neighborhood, this may be due to the differences in the types of water storage containers with *Ae. aegypti*'s larvae that affects the density of the adult mosquito, as was the case with a study conducted in Ecuador by Stewart-Ibarra et al. (42)”. (Lines in the text of 600 to 609).

“In Brazil, when analyzing the correlation between the total number of insects collected and Ae. aegypti house indices (HI) in relation to climatic variables of temperature, humidity, rainfall, among others, regardless of locality, these variables had a direct influence on the distribution and presence of mosquitoes (44,45). Therefore, it is important to highligth that mosquito populations can change, to better adapt to local environmental conditions (46,47)” (Lines in the text of 619 to 625).

10. *Please provide additional information on the proportion of DENV-1 and DENV-2 among human samples in the study area. This data will strengthen the vector findings:*

Additional information was as follows: “Of the 21 dengue cases identified for the project, 12 positive cases were identified by RT-PCR, of which the DEN-1 serotype was the most frequent with 75% (9/12), followed by the DEN-2 serotype with 25% (3/12). The remaining nine were identified by detection of IgM antibodies using the Elisa technique and six of these also had the presence of NS1 antigen.”. (Lines in the text of 669 to 674).

11. *Please also discuss the challenges in conducting entomo-virological surveillance. Even though it is essential, many challenges affect the utility of this approach:*

Lines in the text of 679 to 700:

“These findings underscore the importance of entomo-virological surveillance as part of the routine surveillance of municipalities which is a useful tool to identify high-risk areas for virus transmission and an epidemiological alert system to directly control critical areas of activity for vector control especially because people circulating or living in places where these infected mosquitoes are identified are exposed to bites most of the day (48–51). Strategies for virological surveillance of Aedes mosquitoes commonly focus on females because females are hematophagous, whereas males are not (52). Although DENV is transmitted primarily by the bite of female mosquitoes infected with human blood (horizontal transmission), both vertical (in which the infected mosquito is able to transmit the virus to its progeny) and sexual (when an infected male transmits the virus to the female during copulation) transmission have been suggested as an important mechanism for maintaining arbovirus circulation in vector populations (53–55). However, the challenges for the incorporation of entomo-virological surveillance in dengue control programs must have the necessary infrastructure, trained personnel and political (financial) support to implement it and the importance of evaluating its impact on the disease burden in the short, medium, and long term. Its inclusion in control programs should be assessed according to local capacities and the integrated use of other control tools (56)”.

**Responses to reviewer's comments 2**

1. *The author's report regarding dengue fever should include an explanation of the laboratory test that was used to determine confirmed dengue cases, either by a serological test (NS-1, IgG, IgM) or viral load. The author should also elaborate on which WHO criteria that was used. The author needs to be careful when citing references. For example, in line 178 references number 20 and 21 did not refer to the WHO diagnostic criteria.*

“Laboratory confirmation of dengue virus infection was done through RT-PCTR tests in the case of patients recruited ≤ five days of symptom onset and by detection of IgM antibodies by ELISA in those recruited from day six of symptom onset, in accordance with WHO criteria (22).” The reference was updated. (Lines in the text of 177 to 181).

2. *Rather than describing the result, the authors should explain the different findings in the correlation of temperature, humidity, and precipitation with vector density, in Serranía, 29 de noviembre, and 24 de diciembre. Explain why in Serranía there was no correlation between vector density with temperature, humidity, and precipitation, while on 29 de noviembre, and 24 de diciembre significant correlation was observed.*

“..which has a good water supply all year round”. (Lines in the text of 580 to 581).

*“…*such as the presence of the vector outside the homes such as disposable containers, tires, among others”. (Lines in the text of 583 to 584).

“In these neighborhoods, due to the poor water supply, the inhabitants are forced to store water throughout the year, especially in dry weather”. (Lines in the text of 590 to 591).

“That is why the rains favor the increase of breeding sites and cases of dengue. In dry periods and low temperatures, vectors decrease, a situation that does not stop the transmission of the disease, due to the active biting behavior of the vector that favors the spread of the virus (41). It can be observed that the impact of rainfall varied according to the neighborhood, this may be due to the differences in the types of water storage containers with *Ae. aegypti*'s larvae that affects the density of the adult mosquito, as was the case with a study conducted in Ecuador by Stewart-Ibarra et al. (42)”. (Lines in the text of 600 to 609).

“In Brazil, when analyzing the correlation between the total number of insects collected and Ae. aegypti house indices (HI) in relation to climatic variables of temperature, humidity, rainfall, among others, regardless of locality, these variables had a direct influence on the distribution and presence of mosquitoes (44,45). Therefore, it is important to highligth that mosquito populations can change, to better adapt to local environmental conditions (46,47)” (Lines in the text of 619 to 625).

3. *The authors need to elaborate on the inconsistency between Table 3 and the paragraph preceding the Table. The total cases reported by SIVIGILA in the paragraph preceding the table and table were consistent, but there was inconsistency in the Total cases captured by the project in the paragraph preceding the table which was reported to be 21 while in Table 3 the total cases captured by the project was 33 and the human positive dengue cases was 21. The author also needs to put the laboratory test that was used to confirm dengue infection in these people.*

The paragraph was corrected:

“During this period, 77 cases of dengue fever were recorded, 56 of which were reported by SIVIGILA and the total number of cases captured by the project was 33 and the number of positive dengue cases in humans was 21 in the neighborhoods mentioned, being 14, 5 and 2 cases in Serranía, 24 de diciembre and 29 de noviembre, respectively.” (Lines in the text of 485 to 490).

4. *The author should consider putting the sample size formula in The Sample subsection line 194 so that the study could easily reproduced by others who want to do similar research in different countries.*

The sample size was calculated according to the following formula:

$$=\frac{Z^{2}NP\left( 1-P \right)}{N-1e^{2}+Z^{2}P(1-P)}$$

Where;

Z =1,96 (95%)

N= number of houses in the neighborhood

pq= variance

e= sampling error

(Lines in the text of 203 to 209).

Kind regards;

**Wilber Gómez-Vargas**

Gomez.wilber@uces.edu.co
